# Pulsed field ablation of the premature ventricular contractions originating from the medial free wall of the right ventricular outflow tract infundibulum: a case report

**DOI:** 10.1093/ehjcr/ytaf398

**Published:** 2025-08-20

**Authors:** Łukasz Zarębski, Piotr Futyma

**Affiliations:** Clinical Electrophysiology, St. Joseph’s Heart Rhythm Center, Anny Jagiellonki 17, 35-623 Rzeszów, Poland; Medical College, University of Rzeszów, Warzywna 1A, 35-310 Rzeszów, Poland; Clinical Electrophysiology, St. Joseph’s Heart Rhythm Center, Anny Jagiellonki 17, 35-623 Rzeszów, Poland; Medical College, University of Rzeszów, Warzywna 1A, 35-310 Rzeszów, Poland

**Keywords:** Pulsed field ablation, Premature ventricular contractions, Infundibular part of the right ventricular outflow tract, Case report

## Abstract

**Background:**

Premature ventricular contractions (PVCs) originating from the infundibular region of the right ventricular outflow tract (RVOT) may be challenging to ablate due to thin myocardial wall and proximity to the coronary arteries in this region. In such anatomically sensitive regions, the use of radiofrequency (RF) energy may carry a risk of collateral injury or prove ineffective. We present a case report describing successful ablation of infundibular PVCs using pulsed field ablation (PFA).

**Case summary:**

A 38-year-old female with highly symptomatic, monomorphic PVCs was referred for repeat ablation following a previously ineffective procedure performed with RF energy. Intracardiac mapping localized the earliest ventricular activation to the medial free wall infundibulum of the RVOT. A series of high-power RF applications were delivered at the site of earliest activation; however, elimination of PVCs was not achieved. Given the ineffectiveness of RF ablation, the procedure was continued using PFA. A series of focal-bipolar PFA applications were delivered at the RVOT infundibulum target site. This resulted in complete elimination of PVCs. The procedure was completed without complications. At 4-month follow-up, the patient remained asymptomatic, and 24-h Holter monitoring confirmed the complete absence of PVCs.

**Discussion:**

This case demonstrates the feasibility of focal-bipolar PFA for PVCs arising from the RVOT infundibulum. Pulsed field ablation may offer a safe and effective alternative in anatomically challenging locations, particularly when conventional thermal energy sources are unsuccessful.

Learning points

- Pulsed field ablation (PFA) provides a safe and effective option for premature ventricular contractions (PVCs) originating from the infundibular right ventricular outflow tract (RVOT), offering durable lesion formation while reducing the risk of injury to adjacent coronary arteries and valvular structures.
- The close proximity of the infundibulum to the left anterior descending artery and its thin myocardial wall limits the safety and efficacy of radiofrequency ablation, supporting the role of non-thermal PFA in this challenging anatomical region.

## Introduction

Catheter ablation of premature ventricular contractions (PVCs) originating from the medial free wall infundibular part of the right ventricular outflow tract (RVOT) can be challenging due to the infundibulum’s specific anatomy and proximity to coronary vessels.^1^ Extensive radiofrequency (RF) ablation in this area may fail to create durable lesions and carries a risk of collateral injury.^2,3^ Pulsed field ablation (PFA) is a novel, non-thermal modality that selectively affects myocardial cells while sparing surrounding structures.^4^ We report a case of successful bipolar PFA of the medial free wall infundibular RVOT PVCs following a failed RF attempt.

## Summary figure

**Figure:**
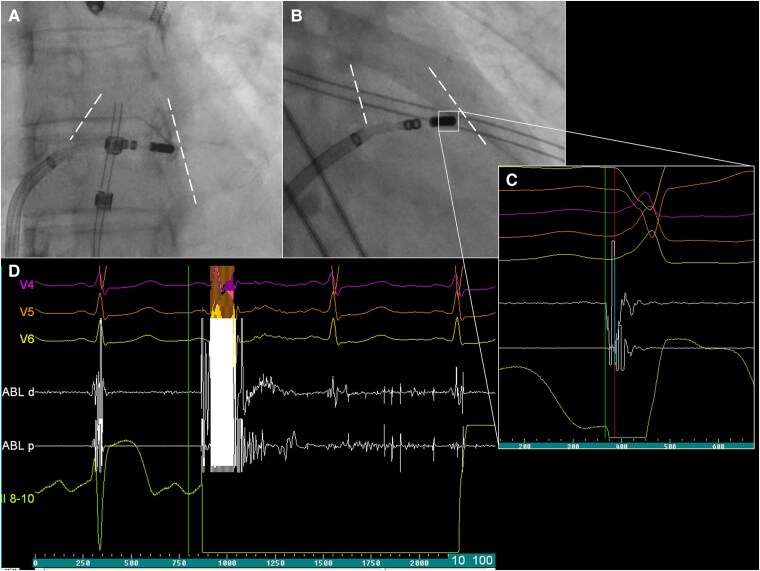


## Case presentation

A 38-year-old female with no structural heart disease was referred for repeat catheter ablation due to highly symptomatic, frequent PVCs. A 24-h Holter ECG demonstrated a PVC burden of 21%. The patient reported frequent and highly symptomatic palpitations. A prior ablation procedure performed 11 months earlier at another centre had targeted the septal region of the RVOT using RF energy, with additional extensive applications delivered from the adjacent left ventricular aspect. Despite these efforts, the procedure failed to achieve suppression of the arrhythmia. Additionally, the patient exhibited high sensitivity to thermal energy applications, which limited the ability to deliver high-power or prolonged-duration RF lesions and may have contributed to the inefficacy of the initial procedure.

**Figure 1:**
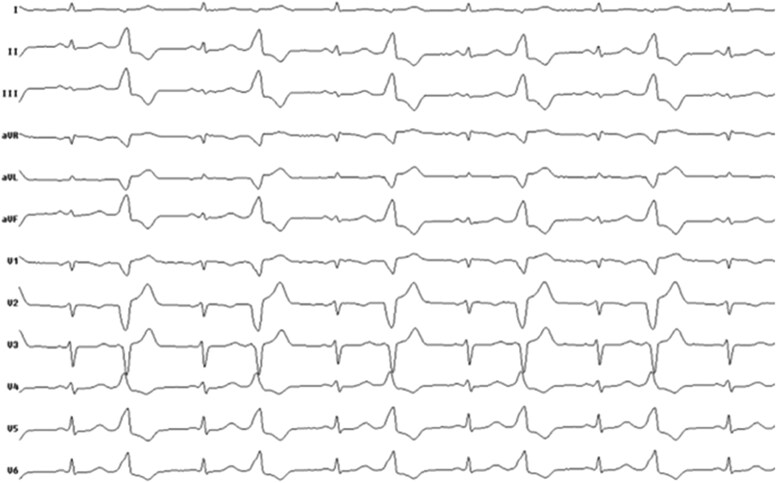
Twelve-lead electrocardiogram with clinical ventricular bigeminy.

**Figure 2:**
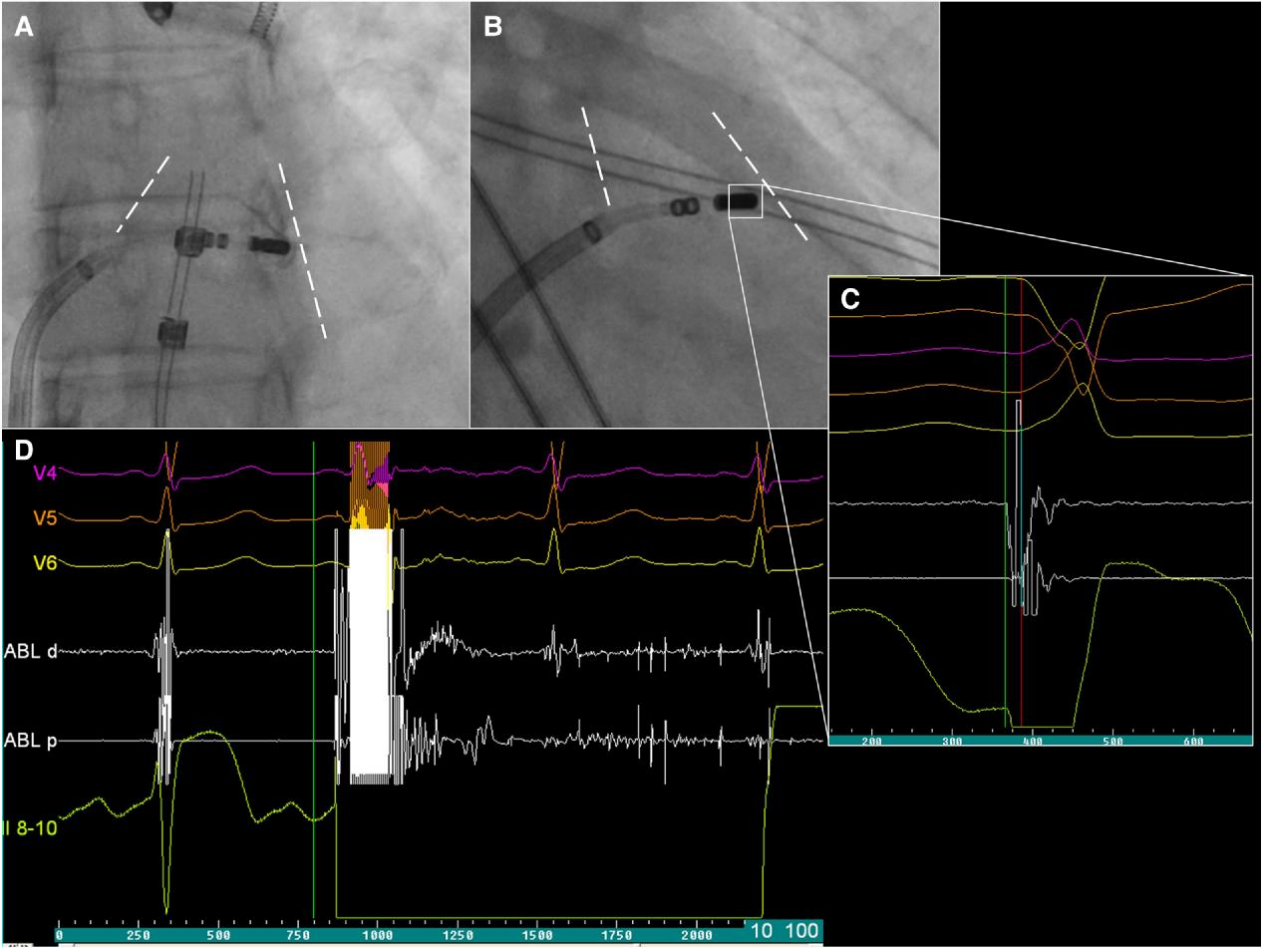
Fluoroscopy images in left anterior oblique (*A*) and right anterior oblique (*B*) projections at the site of an earliest premature ventricular contraction activation (*C*) in the infundibulum region of the right ventricular outflow tract, which is outlined with a dashed line. Focal-bipolar pulse field application (*D*) leading to elimination of clinical premature ventricular contraction. PVC, premature ventricular contraction; RVOT, right ventricular outflow tract.

The repeat ablation was performed under conscious sedation. Vascular access was obtained via ultrasound-guided puncture of the right femoral vein. During the procedure, frequent monomorphic PVCs were observed in a bigeminal pattern (*Figure 1*), with an identical morphology to those documented during the previous ablation attempt. The 12-lead surface ECG demonstrated left bundle branch block morphology with an inferior axis pattern and abrupt precordial transition zone in V4, consistent with an infundibular RVOT origin.^1,5^ Activation mapping localized the earliest ventricular activation at the medial free wall infundibular region of the RVOT (*Figure 2A–C*). Repeat ablation was performed using a 4-mm open-irrigated FlexAbility catheter (Abbott, St. Paul, MN, USA), delivering four RF applications at the target site, with an energy of 50 W. During the initial RF applications, no technical issues were observed; impedance remained stable or showed a gradual expected decrease. However, at the fourth RF application, a sudden impedance rise from ∼110 to 140 Ω occurred shortly after energy delivery began, prompting immediate termination of RF. Given the absence of clinical effect despite impedance drop and anatomical complexity of the ablated region, the decision was made to continue the procedure using PFA, which had previously been considered as a bailout strategy. Preceded with 2 mg of nitroglycerin administration, a total number of four focal-bipolar PFA applications at 2000 V were delivered at adjacent sites within the medial free wall of the RVOT infundibulum (*Figure 2D*) from the same FlexAbility catheter connected to the PFGen Generator (CorSystem), which resulted in immediate and complete elimination of PVCs. During the 15-min waiting period, no clinical arrhythmias were observed. The procedure was completed without complications, and the patient was discharged the following day. At 4-month follow-up, she reported complete resolution of palpitations with 24-h Holter monitoring confirming the total absence of clinical PVCs. The patient was not receiving any antiarrhythmic drugs or β-blockers during the follow-up period.

## Discussion

**Figure 3:**
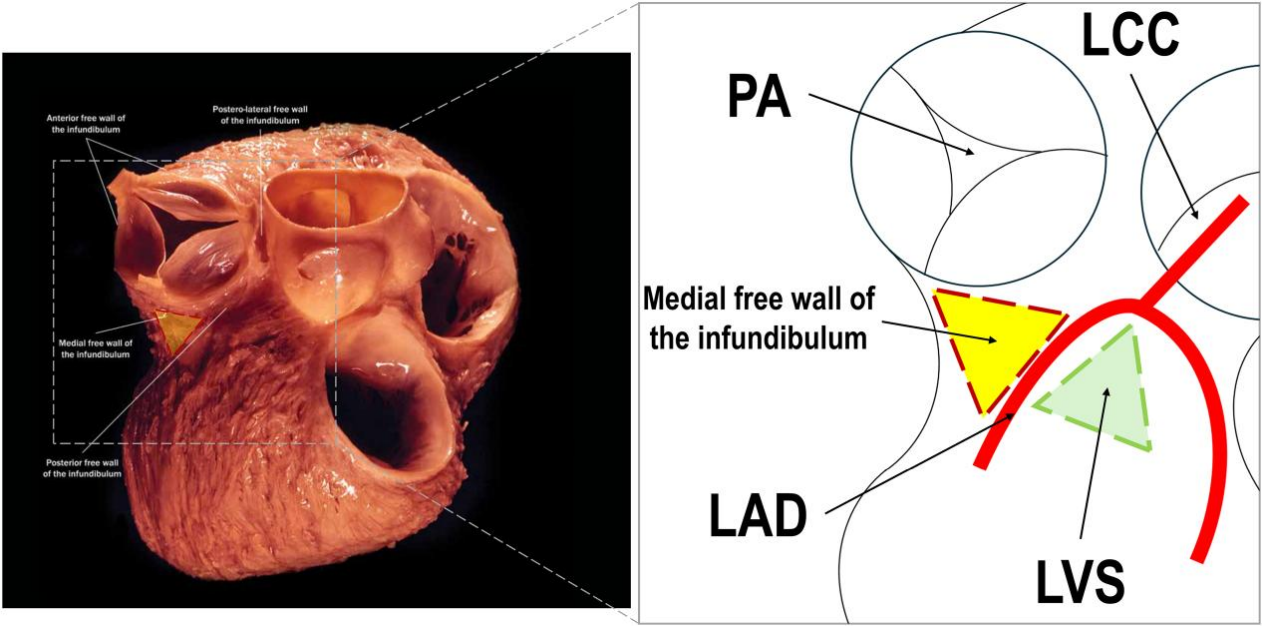
Anatomical relations of the medial free wall infundibulum of the right ventricular outflow tract with schematic illustration of topography in the region. Anatomical image from Amara Yad Project-Wallace A. McAlpine Collection-courtesy K. Shivkumar MD PhD. LAD, left anterior descending; LCC, left coronary cusp; LVS, left ventricular summit; PA, pulmonary artery.

The infundibulum, also known as the conus arteriosus, is the superior and anterior region of RVOT located proximal to the pulmonary valve. Anatomically, it is delineated by the supraventricular crest, which separates the inflow and outflow tracts of the right ventricle. The infundibular myocardium is smooth and non-trabeculated, with a typical wall thickness ranging from 1.5 to 5 mm.^6^ Anteriorly and to the left, the infundibulum lies adjacent to the left anterior descending (LAD), which runs within the anterior interventricular groove (*Figure 3*). Superiorly, it is bordered by the pulmonary valve annulus. Posteriorly and inferiorly, the infundibulum lies next to the membranous interventricular septum and the aortic root, including the right coronary cusp. The anteromedial left ventricular summit is located immediately adjacent to the leftward aspect of the infundibulum,^7^ separated only by a thin layer of the myocardium and fibrous tissue.

Although PVCs originating from the infundibulum are relatively rare, their close proximity to the LAD and the thin myocardial wall can make their successful ablation particularly challenging.^1,6^ These anatomical obstacles may prevent effective RF energy delivery: high-power values combined with increased pressure applied to the tip of ablation catheter can lead to overheating and perforation, while lower settings frequently fail to achieve durable, transmural lesions. Moreover, blood flow in adjacent coronary vessels acts as a ‘heat sink’, cooling the tissue and limiting heat propagation, which in practice prevents attainment of temperatures necessary for irreversible cardiomyocyte injury.^8^ In published series of infundibular PVC ablations, the 1-year recurrence rate reached 27%.^1^ The risk of coronary spasm, or acute LAD damage after RF delivery described in several previous case reports, may further constrain the operator’s ability to deliver sufficient energy safely.^2,3,9–12^

In regions such as the infundibular RVOT, where the anatomical conditions may be challenging, PFA can offer a significant safety advantage over RF energy. Experimental data indicate that coronary arteries exhibit substantial resistance to electroporation, making PFA a preferable option when the ablation site is adjacent to major coronary vessels. Nevertheless, one potential risk associated with the use of PFA near coronary arteries is the induction of coronary spasm that can occur when the field is applied directly to the coronaries.^13^ In the present case, to minimize this risk, nitroglycerin was administered intravenously prior to PFA applications, effectively preventing any evidence of spasm during the procedure. Pulsed field ablation delivered via a standard irrigated-tip catheter allowed for complete elimination of PVCs without complications.

Even though intracardiac echocardiography (ICE) was not used in this case, it could be useful in complex anatomical regions such as the infundibulum of the RVOT closely related to the pulmonary valve and LAD. Intracardiac echocardiography may facilitate more accurate assessment of catheter position and proximity to critical structures. Prior studies have shown that ICE improves the safety of RVOT ablation by enabling real-time visualization of catheter–vessel relationships and identifying structural landmarks not reliably seen with fluoroscopy alone.^14,15^ Its use for PFA cases may be particularly beneficial when applications are performed near coronary vessels.

Several recent studies have documented the use of PFA for PVCs, demonstrating favourable acute success rates and safety profiles. For example, a bicentre study reported on 20 patients treated with PFA for PVCs using the monopolar CENTAURI system combined with Thermocool Smarttouch or TactiCath contact force ablation catheters, achieving an 85% success rate without major complications.^16^ Similarly, another study described 35 patients treated with PFA for PVCs and ventricular tachycardia (VT) originating predominantly from the outflow tracts.^17^ While acute procedural success was satisfactory for both PVCs and VT, favourable long-term clinical outcomes were observed only in patients with PVCs.

Although PFA for PVCs has been already described, most reported cases have used catheters specifically designed or adapted for ventricular ablation. In contrast, our case demonstrates the feasibility of using the FlexAbility catheter originally developed for atrial fibrillation ablation in the ventricular outflow tract. It provided stable tissue contact and allowed precise energy delivery in focal-bipolar mode in the challenging anatomy of the RVOT infundibulum. This suggests that using appropriately designed PFA generator, the FlexAbility catheter may be a feasible option for ventricular arrhythmia ablation with PFA. However, further studies are needed to systematically evaluate its performance and safety profile in ventricular substrates.

## Conclusion

In this case, we described a successful PFA ablation in a patient with PVCs originating from the infundibulum of the RVOT. Our findings suggest that PFA may represent a valuable strategy for treating ventricular arrhythmias originating from anatomically challenging regions such as the RVOT infundibulum.

## Supplementary Material

ytaf398_Supplementary_Data

## Data Availability

All data relevant to this case report are included in the article. Additional data or materials can be made available by the corresponding author upon reasonable request.

## Lead author biography

Piotr Futyma, MD, PhD, FESC, FEHRA (born on October 12, 1983, in Rzeszów, Poland) is an Associate Professor of Cardiology at the University of Rzeszów. After earning his medical degree from the Medical University in Lublin in 2008, he trained in clinical electrophysiology under Prof. Piotr Kułakowski. He pioneered bipolar ablation techniques in Poland and Europe, with landmark procedures published in the *Journal of Cardiovascular Electrophysiology* (2016) and *Circulation Journal* (2018). Author of over 70 peer-reviewed articles (h-index 16), he serves as an Associate Editor *of HeartRhythm Case Reports* and holds editorial roles at multiple journals.

## Supplementary material

**Consent:** The authors confirm that written consent for research purposes was obtained from the patient in accordance with the Committee on Publication Ethics (COPE) guidelines.

**Funding:** None declared.
