# Supplementary figures and images for: Pulsed field ablation of the premature ventricular contractions originating from the medial free wall of the right ventricular outflow tract infundibulum: a case report
Source: Eur Heart J Case Rep. 2025 Aug 20;9(9):ytaf398. doi: 10.1093/ehjcr/ytaf398 (PMC12412440; doi:10.1093/ehjcr/ytaf398)

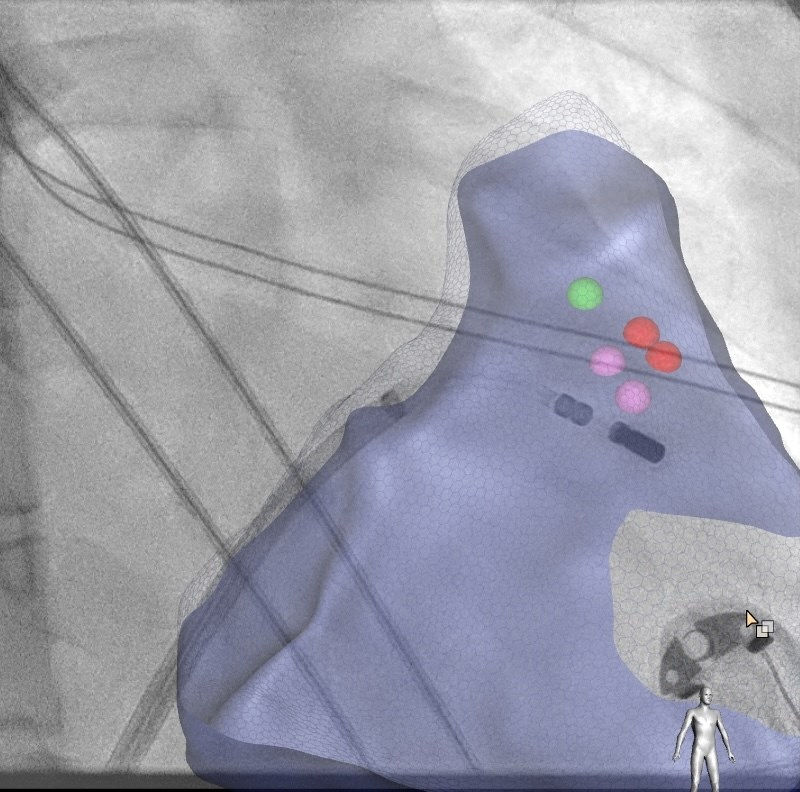

Supplement: ytaf398_Supplementary_Data [file ytaf398_supplementary_data.jpeg]
